# Young Woman With Eye Pain and Blurred Vision While Hair Styling

**DOI:** 10.1016/j.acepjo.2025.100244

**Published:** 2025-08-21

**Authors:** Hideki Fukuoka, Katsura Kobayashi, Chie Sotozono

**Affiliations:** Department of Ophthalmology, Kyoto Prefectural University of Medicine, 465 Kajii-cho, Kawaramachi-Hirokoji, Kamigyo-ku, Kyoto 602-8566, Japan

**Keywords:** corneal burn, thermal injury, hair styling equipment, emergency ophthalmology, ocular trauma

## 1 Patient Presentation

**Figure 1:**
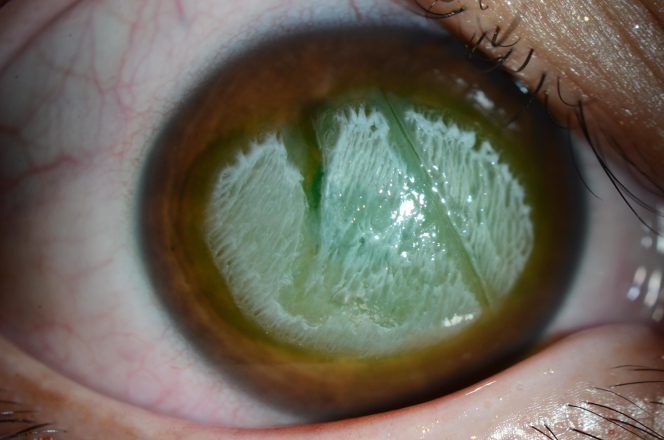
Eye examination showing white-coagulative necrosis of the cornea.

**Figure 2:**
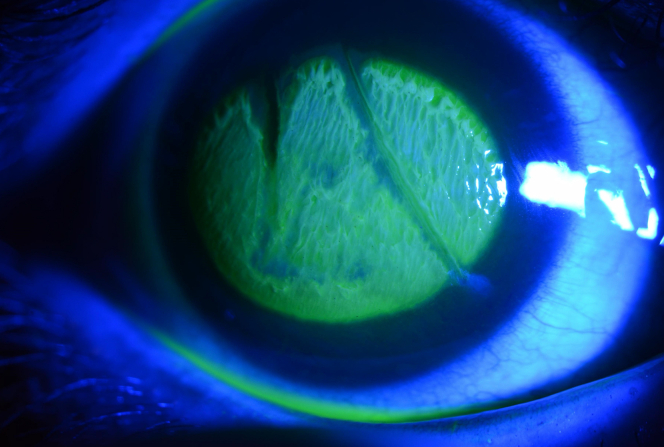
Fluorescein dye, which illuminates areas of corneal injury, demonstrating an approximate epithelial defect of 50%.

**Figure 3:**
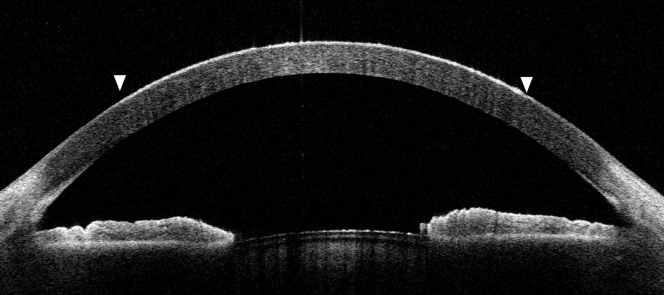
Anterior-segment optical coherence tomography, which provides horizontal cross-sectional imaging of the cornea, showing epithelial irregularity (between white arrowheads).

A 21-year-old female patient presented to the emergency department with acute eye pain and blurred vision after her right eye inadvertently came in contact with a hair iron. The visual acuity of the patient was measured at 20/1000 in the right eye. A slit-lamp examination was conducted, revealing white-coagulative necrosis of the corneal surface, with the corneal-conjunctival junction (limbus) remaining intact, and an approximate epithelial defect of 50% (Figs 1 and 2). Anterior-segment optical coherence tomography, which provides cross-sectional imaging of the cornea, revealed epithelial damage and edema (Fig 3).

## 2 Diagnosis: Burn from Thermal Hair Styling Plate Contact

Thermal hair styling plate-related ocular burns result in immediate coagulative necrosis of the epithelium upon contact, with temperatures reaching up to 200 °C (392 °F). The classic appearance of the condition is characterized by white discoloration, which is indicative of denatured epithelial proteins and associated epithelial defects. The therapeutic approach encompasses the administration of topical antibiotics, steroids, and supportive care measures.^1^

The prognosis is generally favorable when the corneal-conjunctival junction (limbus) remains intact.^2^ The curved corneal surface typically makes only tangential contact with the flat hair iron, thereby sparing the limbus. In this case, the final visual acuity of the affected eye had improved to 20/17. Despite the patient’s initial presentation, which was characterized by significant symptoms, the injuries primarily affected the epithelium rather than the deeper layers of the cornea. This observation is crucial in determining the excellent recovery potential when the condition is managed promptly.

## Funding and Support

By *JACEP Open* policy, all authors are required to disclose any and all commercial, financial, and other relationships in any way related to the subject of this article as per ICMJE conflict of interest guidelines (see www.icmje.org). The authors have stated that no such relationships exist.

## Conflict of Interest

Dr Sotozono reports a relationship with Santen Pharmaceutical Co, Ltd; Aurion Biotech Inc; and Sun Contact Lens Co, Ltd, that includes funding grants. Drs Fukuoka and Kobayashi have affirmed they have no conflicts of interest to declare.

## References

[1] Fish R., Davidson R.S. (2010). Management of ocular thermal and chemical injuries, including amniotic membrane therapy. Curr Opin Ophthalmol.

[2] Dua H.S., Forrester J.V. (1990). The corneoscleral limbus in human corneal epithelial wound healing. Am J Ophthalmol.

